# Euthanasia and assisted suicide for people with an intellectual disability and/or autism spectrum disorder: an examination of nine relevant euthanasia cases in the Netherlands (2012–2016)

**DOI:** 10.1186/s12910-018-0257-6

**Published:** 2018-03-05

**Authors:** Irene Tuffrey-Wijne, Leopold Curfs, Ilora Finlay, Sheila Hollins

**Affiliations:** 1 Kingston University & St George’s, University of London, Cranmer Terrace, London, SW7 0RE UK; 2 Governor Kremers Centre, Maastricht University Medical Centre, PO Box 616, 6200 MD Maastricht, The Netherlands; 3 Cardiff University, Velindre NHS Trust, Cardiff, CF14 2TL UK; 4 St George’s University of London, Cranmer Terrace, London, SW17 0RE UK

**Keywords:** Euthanasia, Physician-assisted suicide, Intellectual disabilities, Autism spectrum disorders, Legislation, Decision-making capacity, Netherlands

## Abstract

**Background:**

Euthanasia and assisted suicide (EAS) have been legally possible in the Netherlands since 2001, provided that statutory due care criteria are met, including: (a) voluntary and well-considered request; (b) unbearable suffering without prospect of improvement; (c) informing the patient; (d) lack of a reasonable alternative; (e) independent second physician’s opinion. ‘Unbearable suffering’ must have a medical basis, either somatic or psychiatric, but there is no requirement of limited life expectancy. All EAS cases must be reported and are scrutinised by regional review committees (RTE). The purpose of this study was to investigate whether any particular difficulties arise when the EAS due care criteria are applied to patients with an intellectual disability and/or autism spectrum disorder.

**Methods:**

The 416 case summaries available on the RTE website (2012–2016) were searched for intellectual disability (6) and autism spectrum disorder (3). Direct content analysis was used on these nine cases.

**Results:**

Assessment of decisional capacity was mentioned in eight cases, but few details given; in two cases, there had been uncertainty or disagreement about capacity. Two patients had progressive somatic conditions. For most, suffering was due to an inability to cope with changing circumstances or increasing dependency; in several cases, suffering was described in terms of characteristics of living with an autism spectrum disorder, rather than an acquired medical condition. Some physicians struggled to understand the patient’s perspective. Treatment refusal was a common theme, leading physicians to conclude that EAS was the only remaining option. There was a lack of detail on social circumstances and how patients were informed about their prognosis.

**Conclusions:**

Autonomy and decisional capacity are highly complex for patients with intellectual disabilities and difficult to assess; capacity tests in these cases did not appear sufficiently stringent. Assessment of suffering is particularly difficult for patients who have experienced life-long disability. The sometimes brief time frames and limited number of physician-patient meetings may not be sufficient to make a decision as serious as EAS. The Dutch EAS due care criteria are not easily applied to people with intellectual disabilities and/or autism spectrum disorder, and do not appear to act as adequate safeguards.

**Electronic supplementary material:**

The online version of this article (10.1186/s12910-018-0257-6) contains supplementary material, which is available to authorized users.

## Background

Views on euthanasia and physician-assisted suicide (EAS) differ widely and are often strongly held. At the heart of the debate is a tension in the weight afforded to key principles of medical ethics: respect for patient autonomy, beneficience, nonmaleficence, justice and scope. These latter two also recognise the role of law in protecting citizens from harm. Patient autonomy underpins patient-centered care, and proponents of EAS cite self-determination as an overriding principle, while opponents highlight the relational nature of human autonomy and point out that self-determination is not without its limits [1]. All agree that a physician’s duty is to relieve suffering. Proponents argue that death relieves suffering, while opponents emphasise that EAS is incompatible with a physician’s duties of beneficence and nonmaleficence.

Interpretation of these ethical principles is reflected in the development of EAS legislation, as well as in arguments opposing such legislation to license doctors to provide lethal drugs under certain specified conditions. The European Association for Palliative Care (EAPC) concluded that it is not possible to achieve consensus on the issue, due to incompatible and clashing normative frameworks [2]. Proponents of EAS in countries without such laws argue that EAS legislation serves to relieve intolerable suffering and promote personal control over dying; that the criteria for EAS laid down by law provide transparency; and that the legal frameworks act as safeguards against illegal practices. Opponents point to difficulties in ensuring that the broadly-worded criteria for legalised EAS are met, to the ongoing higher levels of illegal practices, to the incremental increase of such practices, both legal and illegal [3], and to concerns about how such legislation might affect the safeguarding and rights of vulnerable patient groups [4, 5].

This paper aims to examine and reflect on the way in which legal criteria are applied to patients with intellectual disabilities and/or autism spectrum disorder who request EAS in the Netherlands, the first country in the world to introduce EAS legislation. The authors of this paper are experts in the fields of intellectual disability and end-of-life care. They come from the UK and the Netherlands, two countries where ethical principles are translated into divergent legal frameworks with regards to EAS. The aim of this paper is not to present a common ethical view or to promote a particular side of the EAS argument, but to contribute to the EAS debate by discussing the implications, including the risks, for two particularly vulnerable patient groups, based on factual reports from a country where EAS is a legal possibility.

### Euthanasia and physician-assisted suicide (EAS) in the Netherlands

In 2001 the *Termination of Life on Request and Assisted Suicide Act* (“the 2001 Act”) was passed in the Netherlands, making it legally possible for physicians to terminate the life, or assist the suicide, of a patient provided that six ‘due care’ criteria have been met. These due care criteria include the requirement that the EAS request is voluntary and well-considered and that the patient experiences unbearable suffering without prospect of relief. All decisions by doctors to administer euthanasia or assist a patient’s suicide must be reported to a regional review committee (*Regionale Toetsingscommissie Euthanasie* (RTE)) legally tasked with scrutinising after the event whether the decisions that were made met the due care criteria.

The 2001 Act requires the RTE to issue annual reports describing their findings. These annual reports are available on the RTE website (including English, French and German translations from 2010), along with further selected case summaries (in Dutch only) *(**www.euthanasiecommissie.nl* */* *https://english.euthanasiecommissie.nl*). In 2015, the RTE published a Code of Practice which outlines the issues that the committees regard as relevant in performing their statutory task; this too is available on the RTE website, including an English translation. The Code of Practice gives guidance to physicians on assessing whether a patient meets the criteria for EAS. It acknowledges, for example, that ‘unbearable suffering’ is a subjective notion: what is bearable for one patient may be unbearable for another. It stipulates that the suffering must be ‘palpable’ to the physician and states that “the physician must therefore not only be able to empathise with the patient’s situation, but also see it from the patient’s point of view” [6] (p.14). The case summaries and annual reports are written in lay language. They are explicitly meant to give an impression of how the committees apply and interpret the due care criteria for euthanasia and physician-assisted suicide as set out in the Act, and as such, they serve as ‘case law’. Additional file 1 gives an overview of the practice of EAS in the Netherlands, the due care criteria, and of the RTE.

It is important to note the factors that are *not* mentioned in the 2001 Act. There are no specified causes of suffering for which EAS is permitted, and the Act says nothing about the patient’s life expectancy. In practice, life expectancy will often be limited, but the Act does not rule out granting an EAS request from a patient who may have many years to live [6]. In 2002 the Dutch Supreme Court passed a verdict in the *Brongersma* case (the physician-assisted suicide of an elderly gentleman who suffered immobility and loneliness, and was ‘tired of life’) that the unbearable nature of the patient’s suffering, and the lack of prospect of improvement, must have a medical dimension [7]. From previous verdicts by the RTE, it is clear that the medical condition can be either somatic or psychiatric, and that an accumulation of conditions of old age can also be the cause of unbearable suffering without prospect of improvement. The law also makes it legally possible for patients who are no longer capable of expressing their will (including those with advanced dementia) to have EAS, as an advance euthanasia directive can replace a verbal EAS request. There have been proposals to extend the option of EAS to people who do not suffer on medical grounds but simply feel they have ‘completed life’; in 2016, these proposals were investigated and rejected by parliament [8], but the societal debate and campaign continues. The total number of EAS notifications in 2016 was 6091, which accounts for 4% of all deaths in the Netherlands; of these, over 96% were euthanasia and 3.5% were physician-assisted suicide [9].

### EAS for mental suffering

The number of Dutch cases of EAS for psychiatric problems remains relatively small, but is rising steadily. There were 2 reported cases in 2010, 42 in 2013, and 60 in 2016. EAS for mental suffering has been fiercely debated. Arguments have focused on the difficulty of assessing the patient’s subjective perception and complaints and on the challenges in assessing the patient’s capacity of judgement, which may be impaired by psychiatric conditions such as severe depression [10–12]. Arguments in favour of including both mental and physical suffering as legally acceptable grounds for EAS focus on the fact that a key objective of such legislation is to relieve suffering. On what reasoning can it be offered to people with certain conditions (medical or otherwise) which cause suffering but not to others, given that psychological suffering may be as unbearable as physiological pain? It is worth noting that the reasons often given for EAS requests, even in cases of somatic conditions, are not so much a fear of pain: it is the functional limitations and dependence on others that many people find intolerable to such an extent that they would prefer to die [13].

### EAS, intellectual disability and autism spectrum disorder

People fall within the definition of having an intellectual disability if they have a significantly reduced ability to understand new or complex information and to learn and apply new skills (impaired intelligence), resulting in a significantly reduced ability to cope independently (impaired adaptive and/or social functioning), which begins before adulthood, with a lasting effect on development [14]. Autism spectrum disorder is a complex and usually lifelong developmental disorder that can cause problems with thinking, feeling and language, characterised by persistent difficulties with social communication and social interaction [15]. Although autism spectrum disorders are common among people with intellectual disabilities, not all people with autistic spectrum disorder have an intellectual disability. Asperger’s syndrome, for example, is a type of autism that is characterised by average or above-average intelligence.

We have found no papers to date that are focused on the implications of EAS legislation for people with an intellectual disability or autism spectrum disorder, but there have been discussions of the consequences for vulnerable patient groups. Frost et al. [4] assert that it is impossible for doctors to advise patients regarding the point at which their situation becomes less preferable to being dead. They argue that legalising assisted dying by attempting to establish an absolute right to bodily autonomy may undermine other individual and group rights, and that by creating one class of people for whom life is expendable, that particular view may be extended by society to all groups possessing such attributes (such as permanently disabled people). They fear that there would be a risk to the rights of such vulnerable groups in the form of society being less willing to provide for their care and support.

Krahn [5] reports on a debate by the Disability Section of the American Public Health Association on the meaning of policy on physician-assisted dying for people with disabilities, and concludes that this group bears a disproportionate share of the burden:

“Because aid in dying is used primarily to address loss of autonomy and not pain management, and because proponents argue that severe disability should warrant the right to end their life, this suggests a devaluing of a life lived with significant functional limitations…If society endorses the right of a person to seek physician assistance to end his or her life because of increasing loss of functional autonomy, what does that say about how our society values the lives of people who live with comparable limitations every day of their lives for years on end?” [5] (p.54)

This relates to the question whether the EAS criteria of ‘unbearable suffering’ can be applied to situations where suffering is caused by either lifelong or acquired disability, which is usually seen as a socially determined condition, not a medical one. A human rights argument is used by disability rights activists who fear that EAS stigmatises life with a disability and potentially discriminates against them. However, proponents of the right to die also cite a human rights argument [16]; this includes some disabled people who desire more control in the timing of their death [17].

The question whether people with intellectual disabilities and/or autism spectrum disorder need particular attention with regards to EAS is an important one – whether in relation to debates around putting in place EAS legislation, or in relation to EAS requests from vulnerable persons in jurisdictions that have such legislation in place. The principle that people with disabilities must not be treated differently on the basis of their disability is enshrined in the UN Convention on the Rights of Persons with Disabilities [18], firmly rejecting an approach where disability automatically disqualifies a person from making decisions. However, because of the serious and irrevocable nature of EAS, it could be argued that vulnerable people need special protection. There is thus a moral tension between people’s right to autonomy on the one hand and the need to prevent harm on the other.

The Dutch due care criteria are intended to protect people from harm, whilst upholding their right to autonomy. This paper looks at how effective the due care criteria are in relation to people with intellectual disabilities and/or autism spectrum disorder. The Dutch RTE Code of Practice states:

“Notifications of cases of euthanasia involving patients with an intellectual disability are rare. There are cases where patients with a mild intellectual disability are capable of making a voluntary and well-considered request for euthanasia, and where all the other due care criteria are met. In these cases, particular attention must be paid to the patient’s decisional competence with regard to a request for euthanasia.” [6] (p.28)

The 2016 annual report includes a brief case summary of a man with an intellectual disability caused by tuberous sclerosis (case 2016–03), aimed at demonstrating that it is indeed possible to administer euthanasia to someone with an intellectual disability. Key to this was a capacity assessment by a physician who had specialised in the care of people with intellectual disabilities [9]. Assessing whether someone has the capacity needed to make decisions, which is crucial for the assessment of the due care criterion (a) ‘voluntary and well-considered request’, could be especially difficult if the patient has an intellectual disability and/or autism spectrum disorder. However, several of the other due care criteria could also present particular challenges for these patients, including criteria (b) assessment of a patient’s suffering, (c) the provision of adequate information, (d) deciding together with the patient that there is no reasonable alternative, and (e) obtaining a second opinion from a physician who does not have a current or recent treatment relationship with the patient [6].

The questions we asked were: How do physicians and the Dutch regional euthanasia review committees operationalise the EAS due care criteria for patients who have an intellectual disability and/or autism spectrum disorder? What, if any, are the particular difficulties when the EAS due care criteria are applied to these patient groups?

## Methods

During the 5 years between 2012 and 2016, the Dutch RTEs received a total of 25,930 notifications of EAS; case summaries of 416 of these are included (in Dutch) in a searchable database on the RTE website (www.euthanasiecommissie.nl). There are no case summaries available from before 2012. The first and second authors (ITW and LC) are native Dutch speakers and fluent English speakers. ITW searched the RTE website for cases of EAS involving patients who had an intellectual disability and/or autism spectrum disorder, by using a range of relevant keywords *(“verstandelijke/intellectuele beperking”, “zwakbegaafd”, “lage/verminderde intelligentie”, “autisme”).* This resulted in 11 case summaries. One was excluded because the text said the patient had been assessed for autism but was found not to have it; and one was excluded as it concerned a patient with dementia rather than an intellectual disability. The keyword ‘cognitive disturbances’ *(‘cognitieve stoornissen’)* led to 38 additional results, all of which were read and excluded, as they concerned patients with psychiatric conditions or dementia but not intellectual disabilities. The nine remaining case summaries were translated into English by ITW and read by all authors. They were analysed using directed content analysis [19] with pre-determined coding categories related to due care criteria (a) to (e) (see Additional file 1). The final due care criterion (f) relates to the drugs used to carry out the euthanasia and was reported using near-identical wording in each of the case summaries; this was excluded from our analysis. Because the number of cases was relatively small, no supportive software was used for the data analysis.

## Results

### The patients

Table 1 presents a summary and selected quotes with regards to the patients’ stated characteristics, diagnosis, and due care criteria (a) to (e). There were six women and three men, with ages ranging from patients in their 30s to a patient in her 90s.

**Table 1:** Summary of cases of EAS for people with ID and/or ASD in the Netherlands (2012–2016)

Patient details •Case number •Sex •Age •ID/ ASD	Medical condition	(a) Voluntary and well-considered EAS request Including details on capacity assessment	(b) Unbearable suffering without prospect of improvement	(c) Informing the patient about his/her situation and prospects NB All 9 case reports include a variation on the following sentence: *“From the documents it can be concluded that the physician and specialist have informed the patient sufficiently about his situation and his prospects.”*	(d) No reasonable alternative NB All 9 case reports include the following sentences: *“Cure was no longer possible.”* *“There were no possibilities to relieve the suffering that were acceptable to the patient.”*	(e) Consulting an independent physician (“consultant”)
2013–21 Male 60–70 ASD Self-diagnosed Asperger’s *“Intelligent”*	Neurological and psychiatric conditions since childhood, incl anxiety- and panic disorders, compulsive disorders, depression and excessive worrying Various psychiatric hospitals throughout life; received ongoing support from the mental health team	Patient had talked about euthanasia for years. 8 months before: a psychiatrist diagnosed severe and probably chronic depression with a persistent death wish; advised that before euthanasia, a psychiatrist SCEN physician should be consulted. Physician found *“The patient was of clear mind, intelligent and knew what he was talking about.”* 1 month before: Psychiatrist SCEN physician examined the patient’s capacity. Found to have full capacity. Did not find any indications of depression or other psychiatric disorder.	*“The patient was an utterly lonely man whose life had been a failure. He responded to everything, even the most simple circumstances, with severe panic. He quarrelled with everyone who wasn’t a support professional. The patient had become totally stuck in isolation and found this increasingly painful. He was tired of fighting and could not find rest anywhere. He could not adapt to new situations either. His health was deteriorating and he would have to move into sheltered accommodation. This thought horrified him. It was also very difficult for the patient to have an overview of how to manage his daily life… The suffering was palpably unbearable for the patient.”*	No further details given	*“Treatment with medication didn’t work because the patient could not tolerate any medicines.”* *“The consultant found that the patient’s personality was very poorly emotionally integrated and therapy-resistant.”*	SCEN physician (psychiatrist) 1 visit, a month before euthanasia
2013–22 Female 70–80 *“Mentally retarded”* Scored low on recent IQ assessment However, this was put into doubt by her physicians, who argued that the low IQ score was due to a lack of schooling (i.e. she lacked the skills to do the test)	Extensive psychiatric history. Severe personality disorder. Impulsive nature. Somatic conditions in recent years incl: vertigo, arthrosis, COPD retinitis pigmentosa causing near-blindness.	Patient had asked repeatedly for euthanasia over 2 years. Disagreement among physicians about capacity: physician thought she had capacity but consulted first a psychiatrist (who disagreed) and a SCEN physician (who agreed). The RTE asked *“Given the fact that the report by psychiatrist T. in 2012 stated that the patient lacked capacity with regards to the euthanasia request, and other reports included various statements that the patient had a psychiatric disorder with roots of intellectual disability, or that she had a medical history as a very unstable, intellectually disabled woman, the committee wondered whether the patient had understood what she was signing when she signed her living will. To what extent was the physician of the opinion that the patient’s possible intellectual disability could possibly affect her decision making capacity?”* The RTE requested further explanations from physician and SCEN consultant (but not from the first psychiatrist who had found that the patient lacked capacity). The physician explained: *“The patient’s possible intellectual disability did not hinder her decision making capacity. She could indicate clearly how much she has struggled with her symptoms throughout her life. She had been treated for these for years, with little effect. She could also indicate clearly what the pros and cons of the offered alternatives were for her, thus testing that she not only understood the facts of the information, but she could also weigh them. According to the Appelbaum criteria for capacity, the patient therefore had sufficient capacity… The patient was not an evidently intellectually disabled person: she had good verbal ability and was very well able to express her feelings and opinions.”* The independent consultant *“felt that the context where the patient later called psychiatrist T. ‘an oaf, who doesn’t understand a thing and held things up’, demonstrates insight… The ability to process information rationally was doubtful, but overall, the patient had capacity.”* The RTE concluded that the physician should have asked yet another psychiatrist to confirm her opinion that the patient had capacity, but in the end accepted that verdict of 2 out of 3 positive capacity assessments.	*“The patient’s suffering consisted of an increase in her experiences that people in her immediate environment were influencing her life and making her life impossible. She suffered from her dependence on the care of others and the fact that she could not control her impulses, and that her life was ruled by her emotions. None of the interventions or medication could give her relief.”* *“The patient suffered enormously and this was visible… It was understandable to the physician that the patient wanted to die.”*	*“She indicated that people kept shining lights into her house and that she was disturbed by this. The physician tried to help her understand that the light flashes were related to her eye disease, but the patient could not be convinced.”*	Throughout life, many admissions to psychiatric hospitals. Extensive and varied treatments incl ECT. *“To reduce external stressors, an attempt was made to change the patient’s living situation. She was temporarily admitted to a care centre; in the end, she could not be motivated to move there permanently. Long-term support from a specialist nurse could not reduce the patient’s suffering either.”* Her physician and socio-psychiatric nurse had various conversations and tried to suggest alternative options and treatments. The patient complied and tried the alternatives (further medication treatment and a training programme) before deciding that they did not help and that her situation was hopeless. Physicians agreed that they had run out of options, and the patient’s symptoms were undiminished.	SCEN physician (GP) 1 visit, 7 weeks before euthanasia
2013–51 Female 60–70 Infantile encephalopathy; mild ID	Heart attack (18 years ago) COPD GOLD IV (5 years ago) Heart failure (3 years ago) Osteoporosis Vertebral fracture Stroke (6 months ago) Recurring TIAs	Had talked about euthanasia with a range of carers, professionals and priest. 3 months before: requested euthanasia A psychiatrist assessed capacity; he found *“no indications of depression in a narrow sense and that the patient had decision making capacity. There was no written declaration because the patient could not read or write.”* (No further details are given)	*“Limited mobility, severe fatigue, attacks of breathlessness that were difficult to curtail, realistic fear of suffocating to death, side effects of medication, dysarthria, pain, palpitations, fear for the suffering that awaited her and the hopelessness of her situation. The patient spent a lot of time in bed. She received oxygen. The patient was no longer able to do the activities that she enjoyed. Because of her situation, the patient was increasingly dependent on care.”*	No details given	No details given	SCEN physician 1 visit, 3 days before euthanasia
2014–77 Male 30–40 ASD (Aspergers) Diagnosed 20+ years ago	Mental health problems. Abuse in early childhood. Several suicide attempts	3 years before: patient requested euthanasia but psychiatrist refused due to conscientious objections and belief that treatment was still possible. Referred to another psychiatrist 1.5 years later, to deal with the EAS request. 1 year before: Sectioned and forced to comply with treatment as a condition for professional’s cooperation with his euthanasia trajectory. 1 month before: several Drs agreed that sufficient treatments had been tried and failed. Patient was assessed by 2 physicians who found that the due care criteria had been met. (No mention of capacity assessments)	*“The patient’s suffering consisted of always being busy in his head with thoughts, on multiple and different levels, and not being able to exclude himself from stimuli or thought processes. He found that exhausting. He really wanted to turn off his thoughts and find rest. The patient suffered from the fact that he had a great need for closeness with others whilst he couldn’t maintain long-lasting social contacts. This was because he misjudged interactions and was inclined to behaviour that crossed boundaries. He could react to things in a spontaneous and intense, sometimes extreme, manner. This often led to problems. However, the patient could not learn from these experiences. He was frustrated by his ‘forbidden’ feelings, such as longing for sexual intimacy. He suffered from his continuous yearning for meaningful relationships and his repeated frustrations in this area, because of his inability to deal adequately with closeness and social contacts. His damaged development and the subsequent poor frustration-tolerance and lack of basic feelings of safety contributed to his reduced capacity to learn to deal with the limitations of his illness.”*	No details given	At initial EAS request (3 years before), psychiatrist refused *“because he didn’t think the patient’s situation was without prospect of improvement and there were still treatment alternatives available.”* *“Following investigations, [the 2nd] psychiatrist came to the conclusion that sufficient treatment- and support options had been tried in order to conclude that the patient had experienced little or no improvement of his complaints. The psychiatrist was also of the opinion that there was no other condition that warranted treatment. She thought that there was unbearable suffering with no prospect of improvement, caused by an autism spectrum disorder, and concluded that his euthanasia wish should be processed.”*	SCEN physician (GP) 1 visit, 1.5 weeks before euthanasia
2014–83 Female 50–60 ID and memory problems; placed under guardian-ship 1.5 year ago; had a mentor	Numerous complaints and conditions, both mental and physical including chronic personality disorder. Past 30 years: anxiety; diabetes mellitus; chronic abdominal problems; pancreatitis; obesity; liver steatosis; gastroenteritis/gastritis. 11 years ago: stroke; hemiparesis; cognitive disorders; aphasic disorders. Lower leg amputation. Wheelchair dependent.	The patient’s mentor/guardian was present at all meetings described. Longstanding persistent death wish. Own physician did not want to consider euthanasia request due to complexity of the case. Supported by her mentor, patient registered herself with SLK. Repeated requests for euthanasia to SLK physician and nurse. Physician considered request voluntary and well-considered (no further details given). Assessed by physician and 3 independent consultants. The 1st consultant *“was not sure whether the request was voluntary and well-considered, given the patient’s psychological condition. He could not establish whether the legal due care criteria had been met, due to the complexity of the case and his lack of psychiatric expertise.”* The 2nd consultant (a psychiatrist) *“tested the patient’s capacity in accordance with the Appelbaum criteria. The patient had the ability to make and express a choice, the ability to understand information, the ability to appreciate the situation and the ability to handle facts rationally. The consultant considered her to have decision making capacity*.”	*“Phantom pain, chronic abdominal pain and chronic chest pain. She had decubitus (pressure sores) which she also experienced as painful, and chronic diarrhoea. The patient suffered from her (physical) disability and wheelchair dependence. She could no longer read and couldn’t enjoy anything anymore. Because of her personality, she was in constant conflict with those around her. This drove her family apart.”* *“Her personality also meant that she needed constant support, direction and care, which made it necessary for her to live in a geronto-psychiatric unit. The patient had no control over her life and felt socially excluded. She suffered from the lack of prospect of improvement in her situation and the futility of her life. The patient experienced her suffering as unbearable.”* *“There was no depression, hallucinations and/or delusions. There was a personality disorder, which coloured the case, but was not an impediment to euthanasia.”*	No details given	*“Multiple medication therapies were tried, and it turned out her pain could not be relieved with analgesia. A pain team could not see any further treatment options either. Psychiatrically, the patient was also treated, at outpatient clinics and during various admissions to psychiatric institutions. The patient has cooperated with all treatments, and there were no realistic treatment options left.”*	SCEN physician (GP) 1 visit, 2.5 months before death Verdict: not sure due care criteria have been met SCEN physician (psychiatrist) 1 visit, 1.5 month before euthanasia SCEN physician (geriatrician) 1 visit, 2 weeks before euthanasia
2015–24 Female 60–70 *“Limited coping abilities with possible reduced intelligence and/or cognitive impairments”*	10 year history of tinnitus *“Initially, the condition was bearable, but in recent years the severity of her complaints increased.”*	Patient’s own GP did not want to meet euthanasia request, so patient registered with SLK (14 months before death) Patient was admitted to a mental health centre for hearing-impaired, as a condition of physician considering her euthanasia request in future. Despite progress made at a treatment centre, *“the patient remained focused on her euthanasia wish, partly due to her low level of intelligence.”* 1st consultant found that patient had capacity, but wanted a 2nd opinion. 2nd consultant found that patient had capacity and was *“well able to make decisions.”* The RTE concluded that *“a picture has emerged of a patient who had cognitive limitations or reduced intelligence, and who had developed an anxiety disorder with regards to noises. She did not make her request on a whim. She was fully aware of the meaning and consequences of her euthanasia request and she was consistent in her request. She was not pressurised by those around her.”*	*“Feeling continually plagued by all sorts of different and terrible noises. She was only free of noise when she was asleep in bed. She could not shower, carry out household tasks or tolerate visitors, due to the effect that the noises had on her. She no longer left the house and had become completely isolated from the outside world.”* *“With regards to the unbearable suffering of the patient without prospect of improvement, the consultant noted the following. Initially, the consultant couldn’t hear the patient properly. She spoke very quietly because otherwise she was troubled by the sound of her own voice. In order to understand her better, the consultant went to sit next to her. Then, when he spoke, he saw the patient flinch. That reaction was not acted. Partly because of this reaction, the suffering became palpable for the consultant.”* *“According to the second consultant, the suffering did not seem to be without prospect of improvement, medically speaking, because for years the patient had rejected every offer of treatment, both psychological and psychiatric, because she deemed these to have no positive effect on her complaints or because she did not feel that she was taken seriously. The patient’s personality traits and her intellectual limitations, however, resulted in an inability to benefit from psychological or psychiatric help.”* *“Tinnitus is a physical condition. With her primitive thinking abilities, the patient was focused solely on eliminating the tinnitus completely. Once she realised ‘I will never get rid of it’, her suffering became unbearable and hopeless to her, and she was then only focused on euthanasia.”*	*“She had been sufficiently informed at her own level”*	*“When the physician looked at the patient and spoke with her, he saw her suffering… The only alternative for the patient would be to commit suicide. The patient had already made several suicide attempts. The physician was convinced that the patient would try it again... According to the physician, euthanasia was the only way out for the patient.”* *“During the follow-up visits, it became clear to the physician that for this patient, there were no possibilities to learn to cope with [tinnitus]. The physician contacted the patient’s GP and various other treating physicians (psychiatrists). Partly based on the information they provided, it had become clear to her that the patient had indeed gone through many treatments in the past, but also, that often the wrong treatments had been instigated. It had also become clear to the physician that the patient often wanted to abandon the treatments, and that the treating practitioners had not encouraged her to try and persevere with these treatment(s) a bit longer.”* *“The patient’s personality traits and her intellectual disabilities resulted in an inability to benefit from psychological or psychiatric help.”*	SCEN physician 1 visit, > 3 weeks before euthanasia SCEN physician (psychiatrist) 1 visit, 7 days before euthanasia
2016–03 Male 30–40 ID due to tuberous sclerosis, diagnosed in early childhood	Tuberous sclerosis affecting multiple organs. Several years ago: progressive tumour growth in abdomen; liver metastases	Repeated euthanasia requests from 2 months before death. *“A skilled doctor and a registered healthcare psychologist assessed the patient’s capacity in relation to his euthanasia request. They concluded that the patient demonstrated insight into his situation and his prognosis, and that he had been able to make an independent decision and understood the consequences of his decision. They decided that the patient had decision making capacity.”*	Pain, nausea, difficulty sleeping, fatigue and exhaustion. *“He had become bedbound and dependent on others. He suffered from the prospect of dying of severe nausea and pain due to internal abdominal bleeding or an epileptic fit, without being able to say goodbye to those around him.”*	No details given		SCEN physician 1 visit, 2 weeks before euthanasia
2016–48 Female 90–95 A year previously: diagnosis of long-standing ASD; diagnosis of mild cognitive disorder (slow thought processes, loss of executive function and mental inflexibility); language disorder	Combination of age-related conditions (deafness, visual limitations, arthrosis, osteoporosis)	Patient had talked about euthanasia for 5 years, but her GP did not do euthanasia, so she moved to a different GP. Her doctor and an independent geriatrician considered her capable: *“Despite the sometimes challenging nature of the conversations, the doctor thought that the request was voluntary and well-considered.”* However, the independent SCEN consultant *“didn’t manage to have a proper conversation with the patient, and therefore he found it difficult to judge whether the criteria had been met… The consultant suggested visiting the patient again in the presence of her immediate family…* *In his second report, the consultant concluded… that the criteria had not been met. The consultant was unable to judge whether the patient had decision making capacity and he could not judge whether her suffering had a medical basis, and whether there were reasonable alternatives. He suggested that the patient was seen by a geriatric psychiatrist, in order to judge the presence of possible psychiatric problems.”* Following this, the consultant visited again. *“In his third report the consultant concluded, partly based on the report of the independent geriatric psychiatrist and based on the discussion with the patient, that the due care criteria had been met. He considered the patient to have decision making capacity and the request to be voluntary and well-considered.”* *“She repeated her explicit request several times, and on the day of the death she expressed her euthanasia wish once more. From the conversations with the patient the doctor inferred that she was fully aware of the meaning of her request and her situation.”*	*“Weakness, general decline, physical limitations leading to a loss of mobility and cognitive decline which caused an increase in the communication limitations that were already present due to the autism spectrum disorder. The patient no longer had any interest in the world around her and was no longer able to form social contact. She was avoidant of care and contact. The patient spent her days in isolation in her room and in fact was only occupied by her activities of daily living. She declined any help from others because she wanted to keep doing everything herself – according to rigid rituals – even when that had become almost impossible. She suffered from the increasing loss of control over her life.”* *“Because of the lack of reciprosity in communication and the seeming lack of feelings of empathy from the patient, it was difficult to judge whether and why the patient was suffering unbearably… Although to the consultant, the patient’s suffering was understandable only to a limited degree, the clearly substantiated explanations of the independent geriatric psychiatrist convinced him that patients with an autism spectrum disorder suffer in a way that may not be directly understandable to others.”*	*“She was capable of repeating the information and agreements of previous conversations.”*	No details given	SCEN physician 3 visits: 6 months, 4 months and 5 weeks before euthanasia
2016–73 Female 70–80 “She had a mild intellectual disability”	Chronic schizophrenia (30 years) Difficult life story Psychosomatic problems Post-traumatic stress disorder COPD (Gold 4): 2 years	*“The patient had a euthanasia wish which had existed for longer. However, her GP struggled with the way in which the patient behaved and expressed her wish. The psychiatrist who assessed the patient a year before the death, stated in his report that the patient was able to convince him of her death wish, but not of the consistency of that wish.”* Patient self-referred to SLK; physician and consultant found request consistent and well-considered *“even though it was at the patient’s limited cognitive level”* Doctors found “patient had decision-making capacity” (no assessment details given). RTE concluded: *“The patient had a mild intellectual disability but it was evident that she clearly realised what she was asking for. There was a consistent euthanasia request, which was well-considered at her own limited cognitive level.”*	Long list of physical and psychiatric problems, including: *“She suffered from chronic pain which was resistant to therapies. She was also limited in her freedom of movement.”* *“The patient suffered not only from her physical condition, but also from her psychiatric problems. Because of the increase in her physical problems, she found she had no strength left to bear it. It was more and more difficult to escape her traumatic past. The patient kept thinking about it and found it awful. She suffered from nightmares and medication to improve her sleeping was not effective enough. She didn’t want to increase her medication, as she didn’t want to walk around like a ‘zombie’. And she anticipated that her suffering would only increase if she talked and had long term contact with mental health services.”* *“The patient had limited capacity for coping and had firmly declined any psychiatric treatments that were theoretically still possible.”*	No details given	*“An independent psychiatrist, who assessed the patient a year before the death in relation to her euthanasia wish, was of the opinion that there were still possibilities for treatment, such as sleeping medication or antidepressants. However, these treatment options were declined by the patient…The [2nd independent] psychiatrist, who saw the patient two and a half months before the death, was of the opinion that there were no adequate old or new treatment methods available, due to the substantial, vague and largely unstated psychiatric problems.”* The RTE concluded: *“She had received sufficient and structured support, and in that sense, she had exhausted all avenues of treatment.”*	SCEN physician 1 visit, 5 weeks before euthanasia

#### Intellectual disability and/or autism spectrum disorder

Six patients had an intellectual disability, although none of the case summaries describe the nature or extent of the intellectual disability, nor how it affected the patient’s life. One woman (case 2014–83) was placed under guardianship and had a mentor who was present at the consultations with the physicians and who helped her register with the End of Life Clinic when her own physician declined to meet her euthanasia request. For one woman (case 2013–22), the intellectual disability and associated perceived lack of decision making capacity was noted by a psychiatrist; however, when the RTE asked the attending physician (who had carried out the euthanasia) for clarification, he said that the patient’s low IQ score was probably due to a lack of schooling. Two men had Asperger’s syndrome. One woman (case 2016–48) had an autism spectrum disorder; slowness of thought and mild cognitive disorder were also mentioned, but it is not clear whether this was due to old age, physical decline or intellectual disability.

#### Social circumstances

There was a fleeting reference to the patient’s family in three case summaries (Case 2014–83: “Because of her personality, she was in constant conflict with those around her. This drove her family apart.” Case 2015–24: “The patient’s family responded positively to this possibility of admission [to a psychiatric unit].” Case 2016–48: The consultant “didn’t manage to have a proper conversation with the patient, and therefore he found it difficult to judge whether the due care criteria had been met… The consultant suggested visiting the patient again in the presence of her immediate family.” It was not reported whether this happened.)

None of the other case summaries contained any indication of the existence of family, friends or partners. A mentor was mentioned in three cases. One patient lived in a psychogeriatric unit and one lived alone; the other case summaries lacked information about the living situation, so it was unclear whether a patient lived alone, with family or others, or in a care setting. Previous admission to a psychiatric in-patient setting was mentioned in six cases. Information on other social circumstances was similarly sparse, although there were several references to a patient’s loneliness and social isolation.

### Applying the due care criteria

- *Voluntary and well-considered request*

The EAS request, usually to a patient’s own GP, was initially refused in six cases. Three GPs “didn’t do” EAS. In the other cases, the physician thought that the case was too complex or the due care criteria had not been met. These patients, whose request had been refused, then went to another physician or to the End of Life Clinic.

There was an emphasis in the Dutch case summaries on assessing the consistency of the patient’s choice, with statements such as “she clearly realised what she was asking for” (case 2016–73). Capacity assessments were mentioned in eight case summaries, although five of those contained no detail beyond the fact that the patient was found to have decision-making capacity. In three cases, physicians disagreed or wanted a second opinion about the patient’s decision-making capacity. The use of the Appelbaum criteria [20] (see Additional file 2) was mentioned in two cases. In one of these (case 2013–22), the physician concluded that the patient did not meet one of the Appelbaum criteria (the ability to process information rationally), but that she had “overall capacity”. One consultant, who felt unable to assess capacity and found that the due care criteria had not been met (case 2016–48), later accepted the positive capacity assessment of an independent psychiatrist; no further explanations were given of how this conclusion was reached.

(b) *Unbearable suffering without prospect of improvement*

The case summaries all described the patients’ ‘unbearable suffering’ in order to explain the EAS request. Two patients (cases 2013–51 and 2016–03) had progressive somatic conditions; the nature of their suffering was described in terms of their physical decline and increasing dependence.

Five had extensive, complex and longstanding psychiatric problems, including personality disorders, schizophrenia and trauma resulting from childhood abuse. Four of these had developed additional somatic conditions, reducing their ability to cope. Difficulty in accepting or coping with changes in circumstances was explicitly ascribed to the presence of an intellectual disability or autism spectrum disorder and judged to be a valid cause of unbearable suffering.

The physicians of a woman in her 90s, suffering from age-related conditions (case 2016–48), found it “difficult to judge whether the patient suffered unbearably” and found that “the patient’s suffering was understandable only to a limited degree”. Assessment by an independent psychiatrist was requested; the explanations of this psychiatrist “convinced the patient’s physician that people with an autism spectrum disorder suffer in a way that may not be directly understandable to others”. It was accepted that the patient’s suffering was unbearable “by looking at suffering from the perspective of someone with autism. The loss of control… can cause unbearable suffering for an autistic person.”

For two patients, for whom various psychiatric and somatic conditions were described, the stated suffering appeared to stem from characteristics of autism spectrum disorder itself, rather than from acquired medical conditions. One man (case 2013–21) was said to be unable to adapt to new situations and found it very difficult to have an overview of how to manage his daily life. For another, a man in his 30s (case 2014–77), who had an extensive psychiatric history, the suffering was vividly and strikingly described as being unable to maintain social contacts or have meaningful relationships. The case summary stated that his suffering was “caused by an autism spectrum disorder” and continued that “cure was no longer possible. The treatment was only palliative.” The statement that “cure was no longer possible” seemed to be standard, as it appeared in all nine case summaries; the palliative nature of treatment was mentioned in seven cases.

(c) *Informing the patient about his prospects*

Each of the case reports included the identical statement that “the physician had informed the patient sufficiently about his/her situation and his/her prospects”. However, there was no information about how the patients were helped to understand their situation, or whether any adjustments needed to be made to support understanding. One patient (case 2015–24) was said to have been “sufficiently informed at her own level”. There is an indication in one case (2013–22) that the physician tried and failed to help the patient understand the nature of her symptoms (flashing lights due to eye disease).

(d) *Lack of a reasonable alternative*

Treatment alternatives were mostly described as having been tried and found to be ineffective. All case reports stated that “there were no possibilities to relieve the suffering that were acceptable to the patient”. The key to this statement appeared to be *“acceptable to the patient”.* In several cases, the physicians thought there might be alternatives to EAS. Complying with another treatment was a condition for entering the EAS trajectory in two cases; both failed to improve the patient’s condition. In one case (2016–73) the psychiatrist who assessed the patient in relation to the EAS request felt that EAS was inappropriate as alternative treatments could be tried, but the patient refused. Another psychiatrist was then found who agreed that there were no treatment options left. Patient refusal or non-compliance was a common reason for physicians running out of options, leading them to agree that the situation had become hopeless. This includes a woman who suffered from tinnitus (case 2015–24); her EAS request stemmed from a refusal to cooperate with treatments that might enable her to live with the condition. Her sole focus on euthanasia rather than possible treatments was explained by her physicians as being due to her “primitive thinking abilities”. Her physician “saw her suffering” and thought that, because none of the other choices being offered to relieve the suffering were acceptable to her, “euthanasia was the only way out for the patient”.

(e) *Consulting at least one other independent physician*

An independent physician, often a psychiatrist, assessed whether due care criteria (a) to (d) had been fulfilled. In all cases, this was a SCEN (Support and Consultation on Euthanasia in the Netherlands) physician who had been specially trained to provide such consultation and give a written opinion. These consultants did not know the patient; he or she read the case notes and typically visited the patient once, between 5 weeks and 3 days before the euthanasia was carried out. A typical statement was “The psychiatrist spoke with the patient… he concluded, partly based on the conversation with the patient, that the due care criteria had been met” (case 2014–77). In one case (2016–48) the consultant visited the patient three times over six months, as he was not sure whether the due care criteria had been met. Two SCEN physicians were consulted in case 2015–24 and three in case 2014–83, also because of uncertainty about the due care criteria.

The RTEs accepted the physicians’ assessments of patient suffering, prospects and capacity in all cases. Where there had been doubts or disagreements, they asked the physicians with the positive assessments of due care criteria (a) or (b) for further explanations, but not those with negative assessments.

## Discussion

In a society where EAS is legalised and a clearly viable option in the eyes of a large proportion of the population, as is the case in the Netherlands (where currently around 1 in 25 deaths are through EAS [9]), there should be clear and compelling reasons if a person with an intellectual disability or autism spectrum disorder is to be denied this option.

Our findings are in line with those of Kim et al. [21] who reviewed 66 Dutch case summaries of patients receiving EAS for psychiatric conditions. In a discussion of capacity evaluations in these 66 cases, two of the cases we examined (2013–22 and 2014–83) are described in detail [12]. They conclude that

“the practice of EAS for psychiatric disorders involves complicated, suffering patients whose requests for EAS often require considerable physician judgement. The retrospective oversight system in the Netherlands generally defers to the judgements of the physicians who perform the EAS.” [21] (p.367)

Dutch physicians thus carry significant responsibility in deciding whether or not a patient’s EAS request can be met. Here we discuss the difficulties they face in applying the due care criteria to the situations of vulnerable patients.

- **Voluntary and well-considered EAS request**

The RTE Code of Practice 2015 states that “decisional competence means that the patient is able to understand relevant information about his situation and prognosis, consider any alternatives and assess the implications of his decision” [6] (p12). The RTE highlights the importance of careful capacity assessments if the patient has an intellectual disability [6]. The case summaries have shown how difficult such assessments can be, and it is therefore particularly unhelpful that so little detail is given about how the conclusion was reached that these patients had capacity.

Under Dutch legislation [22], as in many other jurisdictions including the UK [23], capacity is decision-specific. There is a presumption that capacity should be deemed to exist unless there is evidence to the contrary; in other words, the test for capacity involves disproving someone’s competence for a particular decision. Dutch government guidance states that capacity assessments should be based primarily on a patient’s ability to comprehend the decision, rather than on the outcome of the patient’s decision, as focusing on the latter risks the assessor’s norms and values being decisive. However, it goes on to explain that:

“This does not mean that the assessor must put aside his own norms and values completely. In dialogue between the assessor and the patient, their respective norms and values can influence each other; but in assessing decisional capacity, the assessor’s norms and values must not be unilaterally decisive. However, the nature of a patient’s intended decision can be a reason for assessing his decisional capacity.” [22] *(p.7, translated by ITW)*

This guidance seems to recognise the perhaps difficult-to-avoid fact that a physician’s ideas about what is best for the patient may influence the patient’s choices.

The most commonly used and influential model for assessing whether a patient has the capacity to exercise autonomy in making healthcare choices is the MacArthur model, which consists of four abilities (also known as the “Appelbaum criteria”): to *understan*d the illness and treatment-related information; to *apprecia*te the significance of that information; to weigh up options using *reasonin*g and logic; and to *communicate* a choice [20] (see Additional file 2). The Appelbaum criteria were referred to by Dutch physicians and the RTE as their model for capacity assessments. The use of this model for EAS decisions is not without its difficulties. Whilst impairment of decision-making capacity lies on a continuum, the judgment of decision-making capacity is an all-or-nothing concept (either the patient has capacity to take a particular decision, or he doesn’t). Where on the continuum the cut-off point for competence lies, is therefore a matter of physician judgement. Appelbaum [24] has argued that the stringency of capacity tests should vary directly with the seriousness of the likely consequences of the patient’s decisions. In the reported case summaries, it appears that the bar is not set high. In one case, the physician stated explicitly that a patient did not have all four Appelbaum abilities, but she was still deemed to be competent. This “lower threshold view” was also noted by Doernsberg et al. [12] in their review of capacity evaluations of psychiatric patients requesting EAS in the Netherlands. However, for decisions involving EAS, applying capacity laws and guidance is challenging. Legislation and guidance governing capacity assessments have not been written specifically for assessment of EAS requests; indeed, the Mental Capacity Act 2005 (England and Wales) [25] specifically states at Section 62 that nothing in the Act affects the law relating to assisting suicide. Wide variations and inconsistencies have been reported in the way mental capacity in patients requesting hastened death is conceptualised, but many consider that EAS should only be open to those with a high degree of mental capacity to make such a request [11]. In other words, for a life-or-death decision such as EAS, the bar should be set high, with patients meeting all aspects of stringent capacity assessments. Whether the MacArthur model is in fact appropriate for such assessment is open to debate and requires a comparative prospective research study. The model has been criticised for its focus on cognition, understanding and reasoning, ignoring the importance of emotions, values, biographical and context-specific factors [26–29]. A study by Tan et al. [28] showed that competence to refuse treatment may be compromised in patients with anorexia nervosa because of their difficulties with thought processing and changes in values, which was not captured by the standardised tests for competence. Breden and Vollmann [26] argue that in reality, patients often base a preferred choice on emotions, values or intuitive factors rather than on a rational analysis of all the options. This debate is important and may well lead to future changes in the way decision-making capacity is conceptualised and assessed, but at present, the MacArthur model is reflected in legal frameworks as well as guidance to Dutch physicians. Because of the complexities of capacity assessments and the judgement required of physicians, even within the seemingly clear Appelbaum criteria, it is especially important that the specifics of such assessments are described with greater transparency than we have found within the case summaries. Stringent capacity testing and a high bar for decision-making capacity, under the current model, would mean that the patient has all four Appelbaum abilities. We could not find evidence of such stringent testing in the Dutch case summaries. Most contained no detail of how the Appelbaum criteria were operationalised.

The RTE reports put a heavy emphasis on the consistent or repeated nature of an EAS request, but for people with an intellectual disability, the difficulties with decision-making are more commonly in the area of ‘appreciating the significance of the information’ or ‘reasoning with the information and weighing up treatment options’ (see Additional file 2). An example of how a consistent treatment refusal can be wrongly seen as a competent and autonomous decision is given in a UK study of patient safety issues, where a senior hospital consultant initially thought a patient with intellectual disabilities was being clear and consistent in his treatment refusal:

“[The patient] had cancer and needed surgery. I didn’t realise that he didn’t have the capacity to say ‘no’ to the operation. He didn’t want the operation, and I just thought that was that.” [30] (p.98)

It wasn’t until this patient was seen by the hospital’s intellectual disability nurse specialist, who found that the patient did not fully appreciate the consequences of his decision, that he was found to lack capacity; the patient, who would have died without surgery, had the operation after all, following a ‘best interest’ decision.

A persistent request does not necessarily imply capacity; it might even be indicative of a lack of capacity, if the patient’s intellectual disability leads to difficulties in considering or weighing up alternatives. In case 2015–24 (the woman with tinnitus), the consistency of the patient’s EAS request took precedence over her ability to consider alternatives, which the physicians agreed was impaired due to her low level of intelligence. This was not helped by the fact that “often the wrong treatments had been instigated” and “practitioners had not encouraged her to try and persevere with these treatment(s) a bit longer”. If stringent capacity assessment criteria were to be applied in this case, it may well be that the patient’s inability to appreciate the significance of the information in relation to her own situation, and to weigh up treatment options, would render her incapable to make an EAS decision, however persistent her EAS request. In case 2013–22, the patient interpreted light flashes related to her eye disease as “people shining lights into her house” and was disturbed by this; she “could not be convinced” by the physician’s efforts to help her understand the cause of her symptoms. Under stringent capacity testing, such inability to understand the nature of her condition could have led to the conclusion that the patient lacked decisional capacity. As the Dutch government guidance states:

“In assessing whether the patient understands the nature of his health condition, one aspect that must be checked is whether he can see the relevant causal links, or conversely, whether he ascribes his health condition to a cause other than the ‘objectively’ determined one. A lack of insight into his condition is a factor that can be important in assessing decisional capacity.” [22] *(p.12, translated by ITW)*

It is evident from the RTE verdicts that it does not require patients to meet all four Appelbaum criteria. The difficulties many people with intellectual disabilities have in rationally manipulating information with regard to their situation, the consequences of their decision and the possible alternatives make them particularly vulnerable when the bar for capacity assessment is not set high. Of all four Appelbaum criteria, *appreciation* ability is undoubtedly the most difficult to understand and measure. This conclusion was also reached in a systematic review of decision-making capacity of patients with depression, which found that *appreciation of information* was the ability that was most impaired, but difficult to measure within standard capacity tests [29].

Making a “voluntary and well-considered request” is closely related to the concept of autonomy. Autonomy in decision-making is complex for people with intellectual disabilities and not easily achieved. Whether a person is able to have a high level of self-determination is not necessarily dependent on their intellectual ability. A study of 301 adults with intellectual disabilities found that experience and opportunities to make everyday choices contributed significantly to greater self-determination and autonomy; intellectual capacity, on the other hand, was not a significant factor [31]. People with intellectual disabilities generally have less experience of and control over major decisions that affect their lives than those without intellectual disabilities [32], and therefore may lack the necessary skills in making life-changing decisions.

A recognition that people with cognitive conditions may need not only highly skilled capacity assessments but also robust support in the decision-making process is not clearly addressed within the Dutch case summaries. The presence of a ‘mentor’ at patient-physician meetings was mentioned in two case summaries, but family was notably absent. It may be that family and carers were involved but that this was not reported in the case summaries; or that the reporting physicians wanted to be sure that the patient was not under undue influence of family or carers (‘voluntary request’). It seems, however, that for vulnerable patient groups, the perspectives of those in the person’s social circle can be invaluable. In the UK, inquiries into health and social care provision for people with an intellectual disability have found consistently that including and listening to their family and carers is important in ensuring patient safety [33, 34]. The guidelines for testing impairment under the Oregon Act [35] include the recommendation to consult, where possible and with the patient’s consent, the patient’s significant others about their views on the patient’s competence and the patient’s decision. Similarly, the Dutch government guidance on capacity assessments includes the statement that “the decision about capacity is often difficult and should be discussed with the various professionals and carers involved… this is particularly important for serious decisions” [22]. We found no evidence in the case reports that physicians included the perspectives of family and carers who knew the patient well.

(b) **Unbearable suffering without prospect of improvement**

Under the Dutch EAS system, it is up to the physician to decide whether the patient’s suffering is indeed unbearable; this is then verified by the independent consultant. In the 2016 annual report, it is explained that the review committees

“do not re-examine the same issues as the physician who made the original decision. The RTE cannot do this, as the patient is no longer alive.” [36] (p.24)

The RTE therefore scrutinises whether or not the physician *could have reasonably come to a conclusion* about the degree of suffering, but does not decree whether the physician’s verdict is correct. This places the responsibility for assessing suffering solely on the shoulders of physicians. It is important to remember the gravity of the physicians’ decision: in agreeing that suffering is unbearable and without prospect of improvement, they ultimately agree that it is better for the patient to be dead than to live.

There are obvious difficulties with implementing this criterion. Suffering is a deeply subjective experience, and it could be argued that no person can ever truly understand the experience of someone else’s suffering, nor assess whether such suffering is severe enough to make the patient’s life not worth living [37]. Cormack and Fléchais [38] point out that suffering needs to be interpreted in the context of the patient’s personality structure, coping mechanisms and psychosocial environment, and argue that the Dutch requirement of suffering being ‘palpable’ to doctors raises concerns about counter-transference. The word ‘palpable’ is a perhaps inadequate translation from the Dutch *‘invoelbaar’*, which implies that the physician needs to ‘feel’ or ‘imagine’ their patient’s suffering, from the patient’s perspective. The difficulties with this requirement are clear from those case reports where physicians struggled to understand the patient’s perspective. Case 2016–48 (a woman with autism spectrum disorder) is a striking example. The physician visited the patient several times but was unable to understand the patient’s view of her own suffering. He trusted a psychiatrist who told him that the experience of suffering is different for people with autism spectrum disorder. A physician’s ability to empathise and ‘feel’ his patient’s suffering is a guideline specified in the RTE Code of Practice [6], but it seems that because of the patient’s specific disabilities, it was suggested that this was not possible. Physicians’ acceptance that the suffering of patients with particular conditions may not be obvious to them could have the worrying implication that for people with some conditions or disabilities, a different (lower) standard for judging the severity of the suffering is accepted.

It has been made legally clear that a somatic or psychiatric medical condition must be the cause of the suffering that leads to the EAS request, but there is a lack of clarity around the question whether lifelong disability would qualify as a reason for EAS. If the answer is ‘yes’, the implications for people with disabilities could be very serious. In this context, it is of concern that for several of the patients’, suffering was described in terms of their lifelong condition, rather than their psychiatric illness (see, for example, cases 2013–21, 2013–22 and 2014–77). Autism spectrum disorders and intellectual disability, with concomitant difficulties with social communication and relationships, could make it more difficult for patients to cope with the changes that come with ill health and ageing, or to weigh information, or to understand and accept possible alternatives. In the case summaries, the term ‘suffering’ was used to describe the normal variations in behaviour and perceptions seen in people with autism spectrum disorders which are an inherent part of the person. There were no explanations of how a lifelong condition such as autism spectrum disorder could be seen as “no longer” curable, nor how it might be treated palliatively. Statements about the lack of prospect of improvement, such as “intractable symptoms”, “refractory to treatment” and “palliative treatment”, are meaningless in the context of lifelong disability. This raises the prospect of diagnostic overshadowing, where there is a negative bias impacting on a clinician’s judgement regarding co-occurring conditions in people with an intellectual disability or mental health condition, and symptoms due to a specific condition are attributed to another [39].

It seems clear from the Dutch case summaries that the patients were indeed suffering, and that they viewed their suffering as unbearable. When suffering is the result of a progressive medical condition, its assessment may be relatively straightforward. It is not surprising, perhaps, that case 2016–03 (a patient with an advanced tumour) is highlighted in the RTE annual report as an example of someone with intellectual disabilities who could choose EAS. Difficulties arise when the suffering, or the fact that the suffering cannot be relieved, is related to the nature of autism spectrum disorder or intellectual disability itself – as was the case for those who were highly dependent, had difficulties with social functioning, difficulties in coping with social circumstances, or a tendency not to cooperate with treatments.

Assessing the nature of suffering is a key question and has significance for disabled people, whether intellectual or otherwise. As we have seen, EAS requests are often based not on pain but on loss of dignity and autonomy. An unintended consequence of this rationale for EAS is to imply that lives lived with disability must be undignified, and that disabled people must experience suffering, despite assertions to the contrary by people living with long term disability [40]. Numerous reports in recent years have suggested that the lives of people with an intellectual disability are valued less across society, and that their short life expectancy results from inappropriate value-laden decision-making by healthcare professionals [30, 41, 42].

(c) **Informing the patient about his/her situation and prospects**

All patients need adequate information, but informing patients with intellectual disabilities may require considerable effort. It is crucial that the information is given in a format that the patient can understand, if patients with intellectual disabilities are to be properly supported to make decisions of such a serious nature. Even people with mild intellectual disabilities, who are recognised as being capable of making independent and autonomous decisions in some circumstances, may require information presented clearly in pictures or other reasonable adjustments in order to be able to fully understand and appreciate their situation. The lack of detail about how patients were informed about their situation and prospects makes it very difficult to understand how physicians have done this, or what expert assistance they need in the future.

(d) **No reasonable alternative**

The key challenge in relation to this due care criterion is that the conclusion must be reached by the physician *together with the patient.* This implies a level of partnership working of which many people with intellectual disabilities have little experience during their lifetime, making this a difficult criterion to implement. Partnership working and ‘shared decision-making’ are important in achieving patient autonomy and patient-centred care. It requires an excellent relationship between the physician and the patient. For people with intellectual disabilities and/or autism spectrum disorder, especially those who also have long-standing psychiatric problems, building such relationships is likely to require time, and therefore, applying the usual standards and timeframes for EAS trajectories may put vulnerable patient groups at a disadvantage, or even at risk. In several cases, especially those where the patient’s own physician did not want to enter the EAS trajectory, the relationship between the patient and the physician carrying out the euthanasia was fairly recent. Furthermore, several case reports seem to imply that there were frictions between the physicians and patients, especially around the question whether further treatments were possible and reasonable. The patient who called her psychiatrist “an oaf who doesn’t understand a thing” is an example of this (case 2013–22). It is difficult to see how an assessment of the patient’s prospects can be made, *together with the patient*, in such complex circumstances.

(e) **Consulting at least one other independent physician**

As we have discussed above, assessing capacity and supporting autonomy outside an ongoing relationship with the patient can be problematic for people with intellectual disabilities, and these problems will be magnified if there are also mental health issues. It appears, therefore, that the requirement of a second opinion from an independent consultant (responsible for assessing whether the due care criteria had been met) may not be appropriate for people with cognitive conditions. It will often be too difficult to gain sufficient understanding of the patient and come to a fully informed view in just one meeting, no matter how carefully the consultant has studied all the case notes. It could be argued that it is difficult to assess *any* patient’s request in one meeting, whether they have a disability or not, but it seems that people who have difficulty with communicating and relating to others are at a particular disadvantage.

### Limitations

The most obvious limitation is that the case summaries are unlikely to tell the whole story. They are rather brief, with a frustrating amount of ‘standard text’ that sometimes fails to convey the individual nature of each situation. It may well be, for example, that family and carers were involved or that capacity assessments were far more extensive than was reported, but we could only base our assessment on what was written in the case summaries. It was striking that much illuminating detail was included in those case summaries where physicians had been asked by the RTE to give further explanations. It would be highly valuable to conduct in-depth interview-based research with physicians, families and carers who have been involved with EAS for patients with autism spectrum disorder or intellectual disabilities; this would provide crucial further insight into the patients’ character, life story, social circumstances, important relationships, and crucially, the assessment of the due care criteria. It is possible that the concerns we have highlighted could be dismissed if the full facts of the cases were known. However, as Doernberg et al. [12] point out, since these summaries are meant to act as ‘case law’, not including extensive capacity discussions leads to the conclusion that the RTEs do not seem to expect high levels of scrutiny from physicians, nor a high threshold for capacity.

It was not the aim of this paper to establish how prevalent EAS is for people with autism spectrum disorder or intellectual disabilities, and we are unable to conclude how often such cases arise. We found nine relevant case summaries out of 416 (2.2%), which is roughly in line with the prevalence of intellectual disabilities among the population. However, those 416 published summaries represent only 1.6% of the total number of EAS cases over the 5 year period. It may be that the other 98.4% are more straightforward and less likely to involve such complex situations; on the other hand, it is also possible that intellectual disability is under-reported. In several of the case reports, the presence of “mild intellectual disability” was only mentioned in passing, in a description of the patient’s medical history. It is also possible that many physicians do exercise extreme caution when a patient with an intellectual disability or autism spectrum disorder requests EAS, as indicated by the fact that two thirds of the reviewed cases had their initial EAS request refused. A study of outcomes of the 645 EAS requests to the End of Life Clinic during its first year (2012) found that about half such requests were refused. Factors associated with a request being rejected included being single, a psychological condition, and having loneliness or loss of mental capacity [43]. The lack of information on prevalence does not detract from the aim of this paper, however, which was to examine how the EAS due care criteria are (and can be) applied in these cases.

## Conclusion

This paper tried to answer the question whether there are any particular difficulties in applying the Dutch due care criteria for EAS to patients with intellectual disabilities and/or autism spectrum disorders. Following the examination of the Dutch case reports, we conclude that the safeguards, in the form of legal due care criteria, are not easily applied to people with intellectual disabilities or autism spectrum disorder, and that the usual standards could in fact have the unintended effect of leaving vulnerable patients at risk. For disabled citizens to have equal rights (including the right to EAS in jurisdictions where this is a legal option), there must be ‘reasonable adjustments’ in place to ensure that the standard procedures do not leave them at a disadvantage.

Much attention has been focused on the importance of capacity assessments. The RTE Code of Practice [6] and 2016 Annual Report [9] have stipulated that EAS for people with intellectual disabilities is possible, provided that specific attention is paid to the assessment of decision making capacity. From the literature and our examination of nine case reports published on the RTE website, we conclude that assessment of capacity can be extremely difficult people with intellectual disabilities, however mild. It requires a high level of expertise and an intimate knowledge of the patient. There are specialist intellectual disability physicians in the Netherlands, but there was evidence of involvement of such a specialist in only one of the cases (2016–03). We would suggest that a specialist intellectual disability physician should be involved in all cases where someone with an intellectual disability requests EAS, and that all physicians involved in capacity assessments of patients with intellectual disabilities or autism spectrum disorder (including those offering a second opinion) must have enhanced training in this area. The currently used Appelbaum criteria for capacity assessments are not without their critics. The way in which these criteria are operationalised for people requesting EAS, and the question whether these criteria are in fact the most adequate or sufficient, needs urgent further investigation. To what extent the criterion of *appreciating* information is able to cover the complexities, emotions and values associated with making an EAS decision needs further clarification and debate. This is particularly pertinent for people with intellectual disabilities and/or autism spectrum disorders, but may also be highly relevant to patients with mental health problems or dementia.

The challenges in applying the EAS due care criteria to people with intellectual disabilities or autism spectrum disorder go well beyond capacity assessments, however. We are particularly concerned about the implications of the difficulties in assessing suffering in people with intellectual disabilities or autism spectrum disorder. Dependency, functional limitations and difficulties with integration in society are often part of conditions which these groups of people live with all their lives. The Dutch cases raise the possibility that the bar for assessment of intractable suffering is set lower for people with an intellectual disability or autism spectrum disorder than for the general population, by considering their long term disability as a medical rather than a social condition. We found no evidence of safeguards against the influence of the physicians’ own subjective value judgements when considering EAS decision, nor of processes designed to guard against transference of the physicians’ own values and prejudices. We suggest that it is important for physicians to be aware of, and articulate, their criteria for judging the patient’s suffering; and for the people in the patient’s circle of family and carers to be included in the process, and their opinions to be taken into consideration. We also suggest that an important ‘reasonable adjustment’ for patients with intellectual disabilities and/or autism spectrum disorder is an extension of the usual timeframes for EAS trajectories. Such an extended timeframe can help all physicians involved (including those required to give an independent opinion) to take the time to build a relationship of trust with the patient.

We align ourselves to the aim of the EAPC to promote the availability of the best possible palliative care for vulnerable people, including people with intellectual disabilities, in accordance with current professional opinion (including those of physicians, ethicists and lawyers) in each country. It has been recognised that people with intellectual disabilities need special attention to ensure equitable palliative care, as they have specific vulnerabilities that make it much more difficult to assess, interpret and meet their needs [44].

Within the Dutch system, we urge particular caution in cases of EAS requests from people with intellectual disabilities and/or autism spectrum disorder, with the onus on both physicians and the RTE to demonstrate much more clearly how all due care criteria were met. We suggest that there should be ‘reasonable adjustments’ to act as additional safeguards for vulnerable and disabled patients. Helping someone to die is such a serious and irreversible decision that if there is any doubt or uncertainty, even if only from one physician, we suggest that physicians should err on the side of refusal; and that if the EAS is subsequently carried out, the RTE process should include a review of the opinion of physicians who did not think the due care criteria were met. Looking at the international implications, we suggest that any plans to introduce or adapt EAS legislation should include a very careful assessment of whether such legislation includes sufficient safeguards to protect vulnerable patient groups.

Widening the implications even further, we speculate that many of the challenges highlighted in this paper could also be relevant to patients in the general population, and that they are simply more pronounced or extreme for vulnerable patient groups. It is quite possible that people with intellectual disabilities are like the canary in the coal mine, among the first to come up against issues that turn out to be issues for everyone. It may well be that the ability to use rationality and logic when weighing up the EAS option, and thus decision-making capacity in accordance with standard capacity tests, is impaired in most people affected by the emotional turmoil of terminal illness or suffering caused by chronic conditions. Perhaps the difficulties of physicians to appreciate suffering of patients with intellectual disabilities or autism spectrum disorders is in fact indicative of the difficulties in putting themselves into *anyone’s* shoes. We have argued that people with intellectual disabilities who request EAS need particular attention and stringent assessments in order to protect them from harm. The question whether the Dutch due care criteria act as sufficient and effective safeguards for *any* patient, or whether assessments should perhaps be more stringent for all patients, would need further investigation and is beyond the scope of this paper. If more stringency is needed for all patients, then ‘getting it right’ for people with intellectual disabilities will benefit everyone. We welcome further and ongoing debate on the issues raised.

## Additional files

Additional file 1: Overview of the practice of euthanasia and physician-assisted suicide in the Netherlands. (DOCX 16 kb)

Additional file 2: The Appelbaum Criteria for assessing mental capacity. (DOCX 16 kb)

## Abbreviations

ASD: Autism Spectrum Disorder
EAPC: European Association for Palliative Care
EAS: Euthanasia and Assisted Suicide
ID: Intellectual disabilities
RTE: Regional Euthanasia Review Committee *(“Regionale Toetsingscommissie Euthanasie”)*
SCEN: Support and Consultation on Euthanasia in the Netherlands
