# Supplementary material for: Euthanasia and assisted suicide for people with an intellectual disability and/or autism spectrum disorder: an examination of nine relevant euthanasia cases in the Netherlands (2012–2016)
Source: BMC Med Ethics. 2018 Mar 5;19:17. doi: 10.1186/s12910-018-0257-6 (PMC5838868; doi:10.1186/s12910-018-0257-6)
Supplement: Supplementary file 1 — Overview of the practice of euthanasia and physician-assisted suicide in the Netherlands. (DOCX 16 kb) [file 12910_2018_257_MOESM1_ESM.docx]

**Additional file 1**

**Overview of the practice of euthanasia and physician-assisted suicide in the Netherlands**

In 2001 The Netherlands passed a law, the *Termination of Life on Request and Assisted Suicide Act*, creating an exception to the Criminal Code. Under the Code ending another person's life or assisting a suicide was, and remains today, a criminal offence. The 2001 Act created an exception by providing that the Code would not apply where a physician terminated the life, or assisted the suicide, of a patient and where certain 'due care' criteria had been observed.

Under the Act, the physician must:

1. be satisfied that the patient’s request is voluntary and well-considered;
2. be satisfied that the patient’s suffering is unbearable, with no prospect of improvement;
3. have informed the patient about his situation and prognosis;
4. have come to the conclusion, together with the patient, that there is no reasonable alternative in the patient’s situation;
5. have consulted at least one other, independent physician, who must see the patient and give a written opinion on whether the due care criteria set out in (a) to (d) have been fulfilled;
6. have exercised due medical care and attention in terminating the patient’s life or assisting in his suicide.

The 2001 Act charged regional euthanasia review committees *(Regionale Toetsingscommissies Euthanasie [RTE])* with examining notifications of voluntary euthanasia or assisted suicide (EAS) submitted by Dutch doctors and adjudicating on whether the requirements of 'due care' had been observed. There are five such regional committees, each composed of a chairman and secretary, a physician, a lawyer and an expert on ethical or philosophical issues. If a physician assists a suicide or administers voluntary euthanasia, he or she must notify the municipal pathologist of the cause of death and also produce a report for the relevant regional review committee. Where necessary, the committee may request the physician to supplement his or her report either in writing or orally.

The assessment of requests for euthanasia or assisted suicide is assisted by a system known as SCEN (Support Consultation Euthanasia Netherlands). The SCEN network includes physicians trained in the requirements of the 2001 law and in the assessment of requests. It may be accessed by physicians considering a request to provide advice or a second-doctor assessment.

In 2012, the End-of-Life Clinic *(Levenseindekliniek - SLK)* was set up by the Dutch right-do-die society, to offer EAS to patients whose request for assisted dying was first denied by their own physician. The SLK consists of teams of physicians and nurses who visit patients at home to investigate the EAS request; if they find that all due care criteria are met, an SLK physician can carry out the EAS. Their website states: “Although anyone can ask the Levenseindekliniek for assisted dying, the main focus is on those patients whose requests for assisted dying are almost always denied: psychiatric patients, people with dementia, or patients with non-fatal diseases.” *(www.levenseindekliniek.nl/en/)*
