# Supplementary material for: Euthanasia and assisted suicide for people with an intellectual disability and/or autism spectrum disorder: an examination of nine relevant euthanasia cases in the Netherlands (2012–2016)
Source: BMC Med Ethics. 2018 Mar 5;19:17. doi: 10.1186/s12910-018-0257-6 (PMC5838868; doi:10.1186/s12910-018-0257-6)
Supplement: Supplementary file 2 — The Appelbaum Criteria for assessing mental capacity. (DOCX 16 kb) [file 12910_2018_257_MOESM2_ESM.docx]

**Additional file 2**

**The Appelbaum Criteria for assessing mental capacity**

The most widely used model for assessing medical decision-making capacity is the MacArthur model developed by Grisso & Appelbaum [20]. In order to be competent, the patient must have the following four abilities, also known as the *Appelbaum criteria*:

1. to *communicate* a choice
2. to *understand* information relevant to the decision
3. to *appreciate* the significance of the given information about the illness, possible treatment and probably treatment outcomes for his/her own situation
4. to *reason* with the relevant information, engaging in a logical and rational process of making comparisons and weighing treatment options

If any of these four abilities are impaired, the patient may lack the ability to exercise autonomy with regards to treatment choices. The MacArthur Competence Assessment Tool-Treatment (MacCAT-T) consists of a semi-structured interview of approximately 30 minutes, used to measure the components of capacity and resulting in a score. Grisso & Appelbaum have pointed out that there is no objective cut-off point for legal competence or incompetence. Rather, the MacCAT-T must be used in combination with clinical observations, psychiatric or psychosocial history in order to reach a conclusion about the patient’s decision-making capacity.
